# A Replication Study from Chinese Supports Association between Lupus-Risk Allele in *TNFSF4* and Renal Disorder

**DOI:** 10.1155/2013/597921

**Published:** 2013-07-01

**Authors:** Xu-jie Zhou, Fa-juan Cheng, Yuan-yuan Qi, Ming-hui Zhao, Hong Zhang

**Affiliations:** Renal Division, Peking University First Hospital, Peking University Institute of Nephrology, Key Laboratory of Renal Disease, Ministry of Health of China and Key Laboratory of Chronic Kidney Disease Prevention and Treatment (Peking University), Ministry of Education, Beijing 100034, China

## Abstract

A recent phenotypic association study of genetic susceptibility loci in SLE suggested that *TNFSF4* gene might be useful to predict renal disorder in lupus patients. To replicate the association, two single-nucleotide polymorphisms (SNPs: rs2205960 and rs10489265) were genotyped in 814 SLE patients. Correlations between genotypes and *TNFSF4* expression were determined. The stainings of *TNFSF4* in renal biopsy specimens were checked by immunohistochemistry. The SNPs of *TNFSF4* were associated with renal involvement in lupus patients from the Chinese population (*P* values for rs2205960 and rs10489265 were 0.014 and 0.005 in additive model, resp.). An association between risk genotypes and low C3 levels was also observed (*P* = 0.034). Functional prediction suggested that rs2205960 had a regulatory feature. The risk alleles seemingly correlated with lower *TNFSF4* expression. Strong *TNFSF4* expression was detected in lymph nodes and “apparently normal” paratumor renal biopsy but not in renal biopsies from lupus nephritis. In genome-wide expression data, *TNFSF4* was also observed to be downregulated in LN in both glomeruli and tubulointerstitium from kidney biopsies. However, the associations were marginally significant. Our data firstly replicated the association of *TNFSF4* with renal disorder in SLE patients in the Chinese population, which supported that *TNFSF4* may act as a marker of lupus nephritis. The detailed mechanisms of its role in pathogenesis will still be further needed.

## 1. Introduction

Systemic lupus erythematosus (SLE) is a complex and heterogenous autoimmune disease. Patients with SLE can present with combinations of various symptoms, from skin rashes and oral ulcers to life-threatening glomerulonephritis or neurologic disorders. Genetic predisposition is a major factor in susceptibility to SLE, and genetic variation probably contributes to the heterogeneity in the manifestation of this disease. Assessment of the relationship between specific disease-associated alleles and clinically relevant components of SLE will surely provide directions for new experimental strategies aiming to elucidate genotype-phenotype relationships in patients with SLE and possibly for further therapeutic optimizations [1].

We have previously reported on the confirmed association between *TNFSF4* alleles with SLE susceptibility and its interactions with several other SLE loci in increasing disease risk [2]. A recent phenotypic association study of genetic susceptibility loci in SLE suggested that *TNFSF4* gene might predict renal disorder in lupus patients [1]. As it was the first time to link* TNFSF4 *risk allele and lupus nephritis (LN), a further independent replication was needed, especially from the Asian populations due to their higher prevalence of LN than that in the Caucasians. Detailed association with renal manifestations should also be addressed. And, functional assays investigating its significance in lupus risk, relying on or not relying on renal intrinsic mechanisms, will be recommendable.

## 2. Materials and Methods

### 2.1. Subjects

A total of 814 Chinese lupus patients (33.7 ± 12.3 years, 89.3% females) with Han ethnicity living in Beijing were enrolled. All SLE patients met the revised SLE criteria of the American College of Rheumatology. Of these, 558 (68.6%) patients were diagnosed as LN (abnormal amounts of urine protein or clumps of cellular elements called casts detectable with a urinalysis), of which 341 (41.9%) were confirmed by renal biopsy [3]. The study was approved by the Medical Ethics Committee of Peking University. All patients gave informed consent.

### 2.2. Single-Nucleotide Polymorphism Genotyping

Rs2205960 and rs10489265, which were previously reported to be associated with SLE with the strongest significance by different studies, were included in the current subphenotypes study [2, 4]. Also rs2205960 was selected as the proxy SNP in the only reported phenotypic association [1]. TaqMan allele discrimination assays (Applied Biosystems, Foster City, CA, USA) were used to determine the genotypes. Randomly selected genotypes from 40 patients were validated by direct sequencing. Both genotyping success rates and accuracy rates were 100%.

### 2.3. Functional Prediction for Noncoding Variants

The functional single nucleotide polymorphism database (http://compbio.cs.queensu.ca/F-SNP/) was used to predict the functional effects of SNPs, and functional significance (FS) scores were applied for ranking potential deleterious effects of SNPs [5]. Evidence about SNPs in regulation of the gene was also investigated using the ENCODE data [6].

### 2.4. Association of Genotype with Gene Expression

Genevar software was used to determine associations between sequence variation and gene expression (http://www.sanger.ac.uk/resources/software/genevar/) [7]. The sequence variation and gene expression profiling data were from the following datasets: three tissue types (adipose, lymphoblastoid cell lines (LCLs), and skin) collected from 856 healthy female twins of the MuTHER resource; lymphoblastoid cell lines from 726 HapMap3 individuals; three tissue types (adipose, LCLs, and skin) derived from a subset of 160 MuTHER healthy female twins; three cell types (fibroblast, LCLs, and T-cell) derived from umbilical cords of 75 Geneva GenCord individuals. 

### 2.5. In Vitro Blood Cell Isolation and mRNA Quantification by Real-Time RT-PCR

T and B lymphocytes isolation and mRNA quantification were performed as we previously described. Primers for candidate gene *TNFSF4* were F-5′-TTCAGGTATCACATCGGT-3′ and R-5′-CCTTCAGGGAGATGAGAT-3′, and, for reference, GAPDH were F-5′-CCAAAAGGGTCATCATC-3′and R-5′-ATGAGTCCTTCCACGAT-3′. The PCR cycling parameters were 1 cycle at 95°C for 10 min, 40 cycles at 95°C for 15 s, 52°C for 30 s, and 72°C for 30 s followed by a melting curve to determine the specificity of the PCR products. One random sample as the calibrator and no template negative control were included in every plate. Amplification was done in triplicate. Quantification of *TNFSF4* expression was made relative to GAPDH by calculating the differences in *C*_*t*_ (Δ*C*_*t*_) and relative values determined by 2^(−ΔΔ*C*_*t*_)^ [8]. 

### 2.6. Tissues and Immunohistochemistry

Renal biopsy specimens from 11 lupus nephritis patients were used to detect the staining of *TNFSF4* by immunohistochemistry. Of the samples, the numbers of patients were 1, 1, 2, 6, and 1 for type I (with the risk genotype rs10489265 GG), type II (risk GG), type III (1 with risk GG and 1 with protective TT), type IV (2 with TT, 2 with GT, and 2 with GG), and type V (risk GG) lupus nephritis, respectively. Representative blocks of paraffin-embedded tissues were cut at a 4 *μ*m thickness, dewaxed, and rehydrated. Antigen retrieval was performed by microwaving sections in 10 mM citrate buffer (pH 6.0). Endogenous peroxidase was blocked by incubation for 15 min with a solution of 3% hydrogen peroxidase. To block nonspecific binding, sections were incubated in 3% BSA for 30 min at room temperature. Specimens were incubated with mouse anti-TNFSF4 monoclonal antibody (1 : 20; R&D; Monoclonal Mouse IgG1 Clone 159403) at 4°C overnight, followed by incubation with the Dako Envision system (ready to use; Dako). The sections were lightly counterstained with hematoxylin, dehydrated through an ethanol series to xylene, and mounted. Lymph node tissues were included in each immunohistochemical run to verify the specificity of the staining, and negative controls were produced by substituting the primary antibody with phosphate-buffered saline. One normal renal tissue was also used as control.

### 2.7. Differential *TNFSF4 *mRNA Expression in Renal Biopsies in Open-Access Data

For confirmation, the differential *TNFSF4 *expression was checked in LN compared with healthy controls using publically available data from a more recent large-scale genome-wide gene-expression analysis conducted in renal biopsies [9].

### 2.8. Statistical Analysis

Association analysis was performed by the Chi-square tests or logistic regressions. For comparison of continuous variables, two-tailed bivariate correlations and Spearman's coefficient were calculated. Statistical analyses were performed with SPSS 12.0 software (SPSS, Inc., Chicago, IL, USA). A two-tailed *P* value of less than 0.05 was considered statistically significant. For a replication, the *P* values were unadjusted.

## 3. Results

At allele-type level, as data from the Korean individuals reported by Sanchez et al., no association between rs2205960 (*P* = 0.514) and rs10489265 (*P* = 0.262) with LN was observed [1]. Next, a genetic model analysis testing for a dominant, recessive, and additive model for the association was performed. It suggested an additive model for the association between rs2205960 (*P* = 0.014) and rs10489265 (*P* = 0.005) with LN (Table 1), which was consistent to the data from the European-derived lupus patients. Furthermore, a detailed association with renal manifestations was also checked. The clinical parameters included blood pressure, renal function, pathology classification, and immunological indicators (serum antibody and complement levels). An association between genotypes and serum complement 3 (C3) level was observed. The frequencies of low C3 levels for rs10489265 GG, GT, and TT were 36.8%, 37.9%, and 20.5%, respectively (*P* = 0.034). However, no specific associations between *TNFSF4* polymorphisms and other SLE subphenotypes were observed.

**Figure 1:**
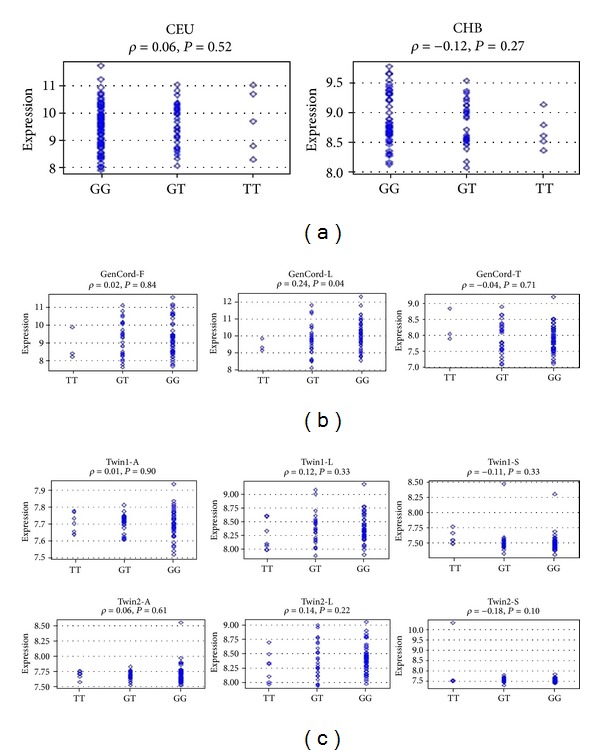
Association between rs2205960 and rs10489265 with *TNFSF4* mRNA expression in cell lines. (a) The expression of *TNFSF4* in transformed B-cell lines from healthy HapMap samples is shown. For space limitation, only data from US individuals with European ancestry (CEU) and Han Chinese individuals from Beijing (CHB) were shown. CEU: 109 Caucasians living in Utah, USA, of northern and western European ancestries; CHB: 80 Han Chinese from Beijing, China. (b) The expression of *TNFSF4* in three cell types (fibroblast, LCLs, and T-cell) derived from umbilical cords of 75 Geneva GenCord individuals is shown. (c) The expression of* TNFSF4* in three tissue types (adipose, LCLs, and skin) derived from a subset of ~160 MuTHER healthy female twins is shown. In MuTHER study, the eQTL analysis was done separately for each tissue. Within each tissue, twins from the same pair were separated by id in two samples analyzed independently. This separation resulted in the following sample size for LCL (L), skin (S), and fat (A), respectively: Twin 1 (74, 76, 79) and Twin 2 (82, 84, 87). SNP-gene association plot is not currently available to 856 healthy female twins of the MuTHER resource in the GENEVAR software. Symbols represent individual subjects. For comparison of continuous variables, two-tailed bivariate correlations and Spearman's coefficient were calculated. Spearman's rho (*ρ*), and nominal *P* value are shown above each plot.

As rs2205960 and rs10489265 were both located in the upstream region of *TNFSF4 *and within one haplotype block with *r* ^2^ 0.96, Thus, their contribution may be derived from gene expression regulatory element. Indeed, by analyzing F-SNP database, it predicted that both rs2205960 and rs10489265 may have transcriptional regulation effect, and the FS scores of them were both 0.101. When the two SNPs were searched in the ENCODE data, the loci for both rs2205960 and rs10489265 were found to lie within regions with enhancer histone marks, DNAse-I hypersensitivity, protein binding, or regulatory motifs in more than one cell type (Table 2). Associations between genotypes and *TNFSF4* expression level were further checked. There are several such expression correlation databases investigating eQTL association patterns within a genetic region of interest available, so the analysis was conducted by GENEVAR, a database and Java tool designed to integrate multiple datasets. Although nonsignificant associations were observed between genotypes of both SNPs and *TNFSF4* mRNA expression (*P* > 0.05) in HapMap3 and MuTHER individuals, it seemed that the risk genotype (rs2205960 TT) was almost associated with lowest *TNFSF4* expression. Of note, in the database from umbilical cords of 75 Geneva GenCord individuals, genotypes of rs2205960 correlated with *TNFSF4* mRNA expression (*r* = 0.234; *P* = 0.041) (Figure 1).

Furthermore, as *TNFSF4* is expressed on the surface of B cells, T cells, dendritic cells, and endothelial cells, B cells and T cells were isolated from 20 healthy controls (numbers for rs2205960 GG, GT, and TT were 9, 8, and 3) and 20 LN patients (numbers for rs2205960 GG, GT, and TT were 7, 11, and 2) to determine their functional significance *in vivo*. No significant differences were observed between LN and healthy controls, as well as among different genotypes (*P* > 0.05, data not shown). 

**Figure 2:**
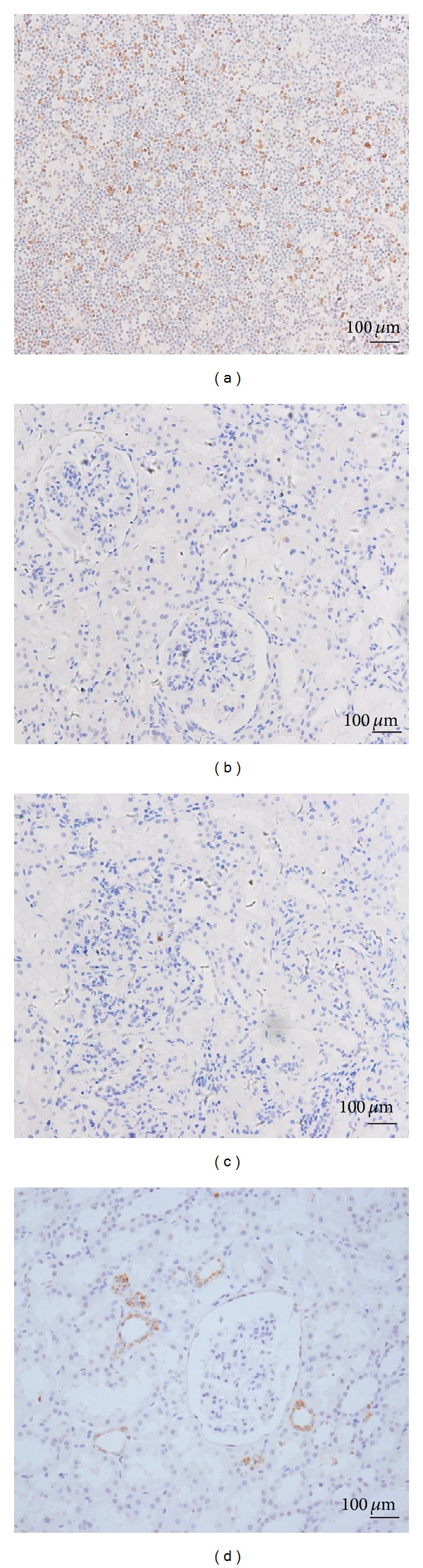
Immunohistochemical stainings of *TNFSF4*. The expression of *TNFSF4* in lymph node (a), renal biopsy from patients with one WHO class IV lupus nephritis (b), negative control (c), and a paratumor renal biopsy without histologic abnormalities (d) were shown. As no obvious expression of *TNFSF4* was detected in glomeruli, no further comparisons were shown according to genotypes of lupus nephritis.

In the lymph nodes, *TNFSF4* was mainly located in the cytomembrane and in the cytoplasm of positive cells in a circular or linear form. In all of the renal tissues including type I, II, III, IV, and V lupus nephritis, no obvious expression of *TNFSF4* was detected in glomeruli, tubules, and vasculature (Figure 2). However, *TNFSF4* expression was detected in a granular distribution at part of tubule epithelial cells from a paratumor renal biopsy without any histological abnormalities. It may indicate less *TNFSF4* expressions in LN kidney than those in “normal tissues”. Indeed, from a genome-wide gene-expression analysis from kidney biopsy which included larger samples, it was observed that *TNFSF4* mRNA expressions were downregulated both in glomeruli (5.61 ± 0.24 versus 5.76 ± 0.18; *P* = 0.044; 32 LN patients versus 14 controls) and in tubulointerstitium (4.64 ± 0.12 versus 4.72 ± 0.14; *P* = 0.052; 32 LN patients versus 15 controls) from LN patients compared with those from controls [9].

## 4. Discussion

Polymorphism at the tumor necrosis factor (TNF) superfamily gene *TNFSF4* was first associated with susceptibility to SLE by both a family-based and a case-control study design [4]. Later, numbers of genome-wide association studies (GWAS) and replications have confirmed such associations [1, 2]. Studies seeking shared autoimmune alleles also suggested that it may be a common risk factor for many other autoimmune diseases. However, a more recent meta-analysis of shared loci between SLE and sixteen autoimmune diseases suggested that it was one of the SLE-specific regions [10]. Although it may be too early to deny its role in autoimmunity, its role as a real genetic locus associated with SLE was conclusively supported. Another important finding was derived from a new study indicating that its risk alleles could predict susceptibility to end-organ manifestations of SLE in the form of nephritis [1]. In the current study, we replicated for the first time the association between *TNFSF4* and the risk of renal disorder in lupus patients from non-European populations. A suggestive association between risk genotypes and low C3 levels, which was an important indicator of disease activity, was also observed. The data will need independent confirmation as they do not survive correction for multiple testing. Our data nevertheless supported that *TNFSF4* may act as marker of lupus nephritis.

Ongoing studies addressing the functional significances associated with individual risk alleles will allow a more precise assessment of its relationship with disease. In the current study, FSNP predicted that the two SNPs may have transcriptional regulation effects. SNP-eQTL analysis supported that risk genotypes correlated with decreased *TNFSF4* expressions. However, the association was not significant from all of the databases, and, in isolated lymphocytes from the Chinese healthy controls and LN patients, such correlation was not confirmed. As the FS score was low, it was, thus, understandable that the relationship between the genotype and expression in lymphocytes was modest. Other reasons may be limited sample size, mixed lymphocyte population *in vivo*, and possible environment or therapy influence. The lack of association between genotypes and *TNFSF4* expression could also be observed from other studies. It may indicate that more samples are needed to determine its functional effect or the genetic variants impact on disease susceptibility by other mechanisms instead of cis-eQTL effect. However, in renal biopsies, less *TNFSF4* expressions in LN than those in lymph nodes and normal renal tissues corresponded to the above speculations. It was strange that a previous study by detecting of binding a recombinant human *TNFRSF4*-containing chimeric molecule did not observe *TNFSF4* expression on any population in peripheral blood at a significant level. But it observed *TNFSF4* rather than *TNFRSF4 *expressed in a granular distribution predominantly along the epithelial side of the glomerular capillary wall, only in type IV lupus nephritis [11]. When the differential *TNFSF4* expression was checked in LN compared with healthy controls using publically available data from a more recent large-scale genome-wide gene-expression analysis conducted in renal biopsies, it was observed that *TNFSF4* mRNA expression was downexpressed in LN in both glomeruli and tubulointerstitium from kidney biopsies. Some reports also identified *TNFSF4* as a biomarker of LN, but they suggested higher *TNFSF4* expression from peripheral blood mononuclear cells [12], which was different from what we observed in B/T cells and renal biopsies. It may indicate discrepancies between systemic effect and cell/tissue-specific effect. Less *TNFSF4* may lead to induction of IL-10-producing CD4(+) type 1 regulatory T (Tr1) cells [13], while higher IL-10 was an intrarenal biomarker of disease activity in lupus nephritis [14]. And an elevated level of *TNFSF4* may significantly accelerate larger atherosclerotic lesions [15], which may deteriorate renal lesions in certain circumstances. For the power and statistics, we observed just multiple lines of association with moderate or marginal associations. The reason may be the underpowered sample size. However, the sample of LN in our study was homogeneous in ethnicity, proven by biopsy, and all enrolled from a single center; the selected tSNP could efficiently tag common alleles of *TNFSF4 *gene and was from GWAS reports; the functional data were checked with external validation using open-access data from other studies, all of which guaranteed reliability. Thus, *TNFSF4* may have both renal dependent and independent roles in pathogenesis of LN. The significance of *TNFSF4* in renal pathology and the relationship between the genotype and expression in the renal tissue are warranted to be further determined. The detailed mechanisms of its role in SLE pathogenesis will still be further needed. 

## 5. Conclusions

In conclusion, our study provided further potential evidence for *TNFSF4* as a risk factor contributing to renal disorder in SLE. The risk alleles may correlate with lower *TNFSF4* expression, whereas LN patients may have less *TNFSF4* in kidneys. The detailed mechanisms of its role in pathogenesis will still be further needed.

## Figures and Tables

**Table 1:** Frequency of *TNFSF4* risk alleles and genetic model analysis for lupus nephritis association in the current study.

SNP (risk allele)	Frequency (%)				Allele level		Genetic model (LN versus non-LN)		
	SLE (*n* = 814)	Non-LN (*n* = 558)	LN (*n* = 256)	Control (*n* = 722)	*P* SLE versus control	*P* LN versus non-LN	*P* Recessive	*P* Dominant	*P* Additive
rs2205960 (T)	34.3%	33.2%	34.9%	27.4%	4.20 × 10^−5^	0.514	0.130	0.061	0.014
rs10489265 (G)	34.8%	32.8%	35.7%	28.7%	2.59 × 10^−4^	0.262	0.204	0.017	0.005

**Table 2:** ENCODE annotations of regions for *TNFSF4* rs2205960 and rs10489265.

SNP	Enhancer histone marks*	DNAse-I hypersensitivity*	Protein binding	Regulatory motifs
rs2205960	GM12878	GM12878, Th1, GM12864	BATF, BCL11A, MEF2A, NF-*κ*B	
rs10489265	HSMM	AG10803, HFF-Myc		GR, Hdx, RXRA, STAT

*ENCODE cell types that show enhancer histone or DNAse-I hypersensitivity regions that overlap SNPs.

GM12878: B-lymphocyte, lymphoblastoid; GM12864: B-lymphocyte, lymphoblastoid; HSMM: skeletal muscle myoblasts; AG10803: abdominal skin fibroblasts; HFF-Myc: foreskin fibroblast cells expressing canine cMyc.

Regulation annotations were identified using HaploReg.
